# Variation in medical claim denials: safety-net providers are hardest hit

**DOI:** 10.1093/haschl/qxag206

**Published:** 2026-08-07

**Authors:** Gabe G Weinreb, Bruce E Landon

**Affiliations:** Health Policy and Management, Harvard Business School, Boston, MA 02163, United States; Division of General Internal Medicine, Beth Israel Deaconess Medical Center, Boston, MA 02215, United States; Department of Health Care Policy, Harvard Medical School, Boston, MA 02115, United States

**Keywords:** managed care, claim denials, reimbursement, safety-net provider, medicaid, medicare advantage, insurance, equity

## Abstract

**Introduction:**

Medical claim denials impose substantial costs on providers but may also reduce low-value care, resulting in lower expenditures and better quality. Little is known about how denial rates vary across payer and provider segments, including for safety-net providers.

**Methods:**

Using a large, multipayer claims dataset from 2019, we examined initial denials, appeal patterns, and final denials by payer segment, service type, and provider safety-net status.

**Results:**

Medicaid managed care had the highest initial denial rate for professional claims (15.1%), while Medicare Advantage had the highest rates for inpatient (20.0%) and outpatient (16.5%) claims. Safety-net providers faced uniformly higher initial denial rates than nonsafety-net providers across professional (13.6% vs 9.2%), inpatient (18.3% vs 14.7%), and outpatient (13.3% vs 12.9%) services.

**Conclusion:**

Safety-net providers absorbed a disproportionate share of denial costs. These providers’ lack of negotiating leverage to secure favorable terms with payers could be 1 driver of these disparities, as could higher rates of low-value care delivery. A standardized framework for the terms and conditions governing payer–provider contracts could help ensure all claims are decided based on the same set of rules.

## Introduction

Medical claim denials have become a flashpoint in debates about the US healthcare system.^1-3^ Denials and subsequent appeals impose substantial financial and administrative costs on providers and may limit patient access to care, but may decrease healthcare spending without adversely impacting quality by discouraging inappropriate or low-value care. Denials are common across several markets, including Medicare Advantage (MA), commercial, and managed Medicaid plans. While comprehensive data on Traditional Medicare (TM) denials are not available, surveyed providers reported that 8% of TM claims were initially denied, compared to 14%-17% for private payers.^4^ Notably, TM has long exhibited large variation in care utilization, and there are persistent concerns about overuse and fraud in TM, highlighting the central tradeoff inherent in claim denials.^5,6^ New federal efforts to apply managed care innovations, such as prior authorization to TM, could help address these concerns.^7^

Claim denials have drawn increasing attention from policymakers and the public. Multiple class-action lawsuits allege that national payers are using automated, computerized algorithms to wrongfully deny claims,^8,9^ and Congressional investigations have scrutinized claim denials in MA, especially for postacute care.^10^ Appealing denials is costly: 1 study found that appeal costs equaled roughly half the denied revenue.^11^ As a result, many denied claims are never appealed.^11-13^ Moreover, the costs of appealing denials fall largely on providers, while the benefits of lower spending and the discouragement of inappropriate care that result from denials accrue to payers and purchasers of care. Though denials primarily affect providers, patient access may also be impacted. For example, several large health systems have exited payers’ networks during contract disputes, citing high denial rates.^14^ Research also suggests that higher denial rates in Medicaid discourage providers from accepting the program, even beyond the impact of low rates.^11^

Despite the importance of claim denials, little is known about how denial rates vary across different types of providers. Understanding how denials affect safety-net providers is important for 2 reasons. First, safety-net providers operate on thinner financial margins, making them vulnerable to a self-reinforcing cycle in which denial-related revenue losses limit investment in the billing and appeals capabilities that would reduce future denials.^15,16^ Second, denials may result in fewer resources for hospitals to invest in programs to improve quality or access. Because safety-net providers disproportionately serve disadvantaged populations, this would exacerbate existing health equity concerns.

In this study, we sought to identify variation in denial rates and appeal patterns by payer, provider, and service type by comparing denial rates for MA, Medicaid, and commercial health plans, and between safety-net and nonsafety-net providers. We stratified all analyses by service type (professional, inpatient, or outpatient) to provide information on variation in denial patterns by setting.

## Study data and methods

### Data

We analyzed 2019 multipayer claims data from Inovalon, a commercial data vendor. Inovalon receives claims data from health plans that use its data analytics and quality measurement services. Over 150 health plans participate, including commercial, MA, and managed Medicaid organizations of varying size across all states of the United States, covering over 95 million enrollees each year. The database contains all services billed to insurance except for postacute care. Many participating plans report claim approval status and include multiple submissions over time, allowing us to track the claim adjudication process.

We used 2 other sources of data to characterize the safety-net status of providers. We used 2019 Medicare claims and administrative data for a random 20% sample of beneficiaries across TM and MA to characterize the patient population of individual clinicians according to the percent of their patients that were dually eligible for Medicaid. The other data source was the 2020 AHRQ Compendium of US Health Systems, a dataset with financial and operational information on over 6700 hospitals compiled from several sources, including financial measures derived from Hospital Cost Reports (see below).^17^

We included in our study professional, inpatient, and facility-based outpatient claims. Ten percent of claims were missing claim payment status. In addition, 25% or more of professional, inpatient, and outpatient claims could not be linked to either the Medicare data or the AHRQ data, and these also were excluded. Excluded claims of these types did not differ from included claims. Additional detail on sample construction is in Appendix Table S1.

### Measures

#### Claim disposition and price

We categorized claims as initial denials if any submission marked them as denied and initial approvals if no submission marked them as denied and included an associated payment amount. If a claim was initially denied but had 1 or more additional submissions, we categorized it as an appealed claim. Appealed claims without any submissions marked as paid were categorized as denied on appeal, and appealed claims with a paid submission were categorized as paid on appeal. Final denials include both never-appealed and denied-on-appeal claims. To calculate spending, we standardized the price for each service to its reimbursement rate in TM. Thus, the prices used to calculate our spending measures reflect Medicare prices and do not vary over insurance segments and carriers.

#### Identifying safety-net providers

For professional services, we used Medicare data to characterize the patient populations of physicians and allied health professionals in the Inovalon dataset. Using Medicare claims, we calculated NPI-level shares of dual-eligible claims, restricting to NPIs with at least 20 claims. We categorized NPIs in the top quintile of dual-share as safety-net providers, following the definition used in the Medicare Quality Payment Program.^18^

For institutional services, we used the Disproportionate Share Hospital Patient Percentage (DPP) from the AHRQ Compendium to characterize hospitals. The DPP measures a hospital's proportion of Medicaid patients and is commonly used to define safety-net hospitals in research and government programs.^17,19,20^ Hospitals in the top DPP quintile were defined as safety net.

### Statistical analysis

We stratified claims by payer segment, service type (professional, inpatient, or outpatient), and provider safety-net status. For each stratum, we report the overall rate of initial claim denials broken down into 3 types: never appealed, denied on appeal, and paid on appeal. Rates were weighted by the Medicare-standardized prices. We also report 3 ratios of these measures: The *appeal rate* is appealed claims as a fraction of denied claims, the *win rate* is the fraction of appealed claims that were ultimately paid, and the *overturn rate* is the fraction of initially denied claims that were ultimately paid. The overturn rate equals the appeal rate times the win rate.

Safety-net and nonsafety-net hospitals have different payer mixes by definition, so differences in denial rates between provider groups could reflect differences within payer or differences in payer mix. In addition to the raw denial rates based on observed payer mix, we present “standardized” denial rates for each provider group that reflect the average payer mix of *all* providers. These standardized rates isolate the within-payer differences between provider groups with the residual representing the across-payer variation.

### Limitations

This study is subject to several limitations. First, payment status codes were missing for 10% of claims. The decision to submit payment status to Inovalon is made at the plan level, so the exclusion of certain plans should not bias the differences in denial rates that we observe between safety-net and nonsafety-net providers. Unless the nonreporting plans have systematically higher or lower denial rates than reporting plans, it also should not bias overall estimates of denial rates or differences between payers. Second, we could not observe the reasons for denials. Third, our data are from 2019 as we only had access to data through 2020, and 2020 data may have been impacted by the COVID pandemic. Fourth, we could not observe the degree to which claim denials discouraged providers from performing certain services at all. Finally, we did not have access to postacute care claims, another potentially significant source of denials.

## Results

Our final sample included 234.2 million claims representing $34.2 billion in spending at Medicare-equivalent prices (Table 1), including 60.2% ($20.6 billion) for professional claims, 13.8% ($4.7 billion) for inpatient claims, and 26.0% ($8.9 billion) for outpatient claims. Categorized by spending, 59.9% ($20.5 billion) was from commercial payers, 29.6% ($10.1 billion) from Medicaid, and 10.2% ($3.5 billion) from MA insurers. Across payers, 21.4% ($7.3 billion) of claims were submitted by safety-net providers.

**Table 1:** Claims and spending by payer and provider type.

	Professional	Inpatient	Outpatient
**Total**	$20.6B (206.4M)	$4.7B (499.3K)	$8.9B (27.3M)
**Payer type**			
** Commercial**	$12.8B (129.1M)	$3.1B (345.0K)	$4.6B (12.4M)
** Medicaid**	$5.7B (57.8M)	$0.9B (93.3K)	$3.5B (12.5M)
** MA**	$2.0B (19.6M)	$0.7B (60.9K)	$0.8B (2.3M)
**Provider type**			
** Safety-net**	$4.3B (43.5M)	$1.1B (107.9K)	$1.9B (6.2M)
** Nonsafety-net**	$16.3B (162.9M)	$3.6B (391.4K)	$7.0B (21.1M)

Source: Authors’ analysis of Inovalon claims dataset.

Spending amounts calculated based on Traditional Medicare prices.

### Denial rates by payer

**Figure 1:**
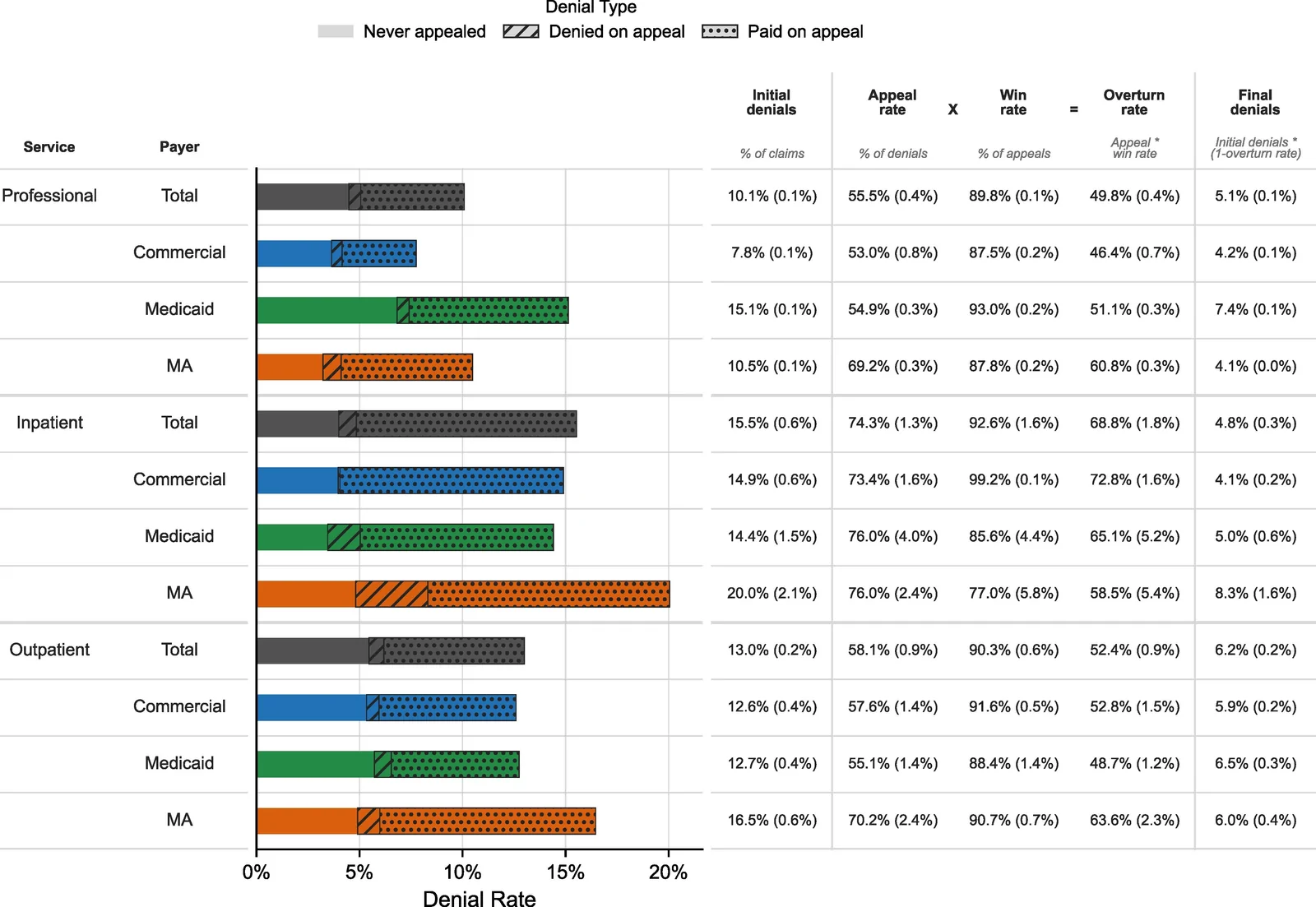
Denial rates by payer type. Source: Authors’ analysis of Inovalon claims dataset. Notes: Spending amounts calculated based on Traditional Medicare prices.

For professional services, Medicaid had the highest initial denial rate at 15.1% vs 10.5% for MA and 7.8% for commercial (Figure 1). Medicare Advantage had the highest overturn rate at 60.8% vs 51.1% for Medicaid and 46.4% for commercial. Thus, despite MA having a higher initial denial rate than commercial, it had the lowest final denial rate (4.1% vs 4.2% for commercial and 7.4% for Medicaid).

For inpatient services, MA had the highest initial denial rate at 20.0% vs 14.9% for commercial and 14.4% for Medicaid. Compounding this, it also had the lowest overturn rate (58.5% vs 72.8% for commercial and 65.1% for Medicaid). Its final denial rate was 8.3% vs 4.1% for commercial and 5.0% for Medicaid. For outpatient claims, MA again had the highest initial denial rate at 16.5% vs 12.6% for commercial and 12.7% for Medicaid. However, MA had the highest overturn rate (63.6% vs 48.7% for Medicaid and 52.8% for commercial). Medicaid had the highest final denial rate (6.5% vs 5.9% for commercial and 6.0% for MA).

### Denial rates by safety-net status

**Figure 2:**
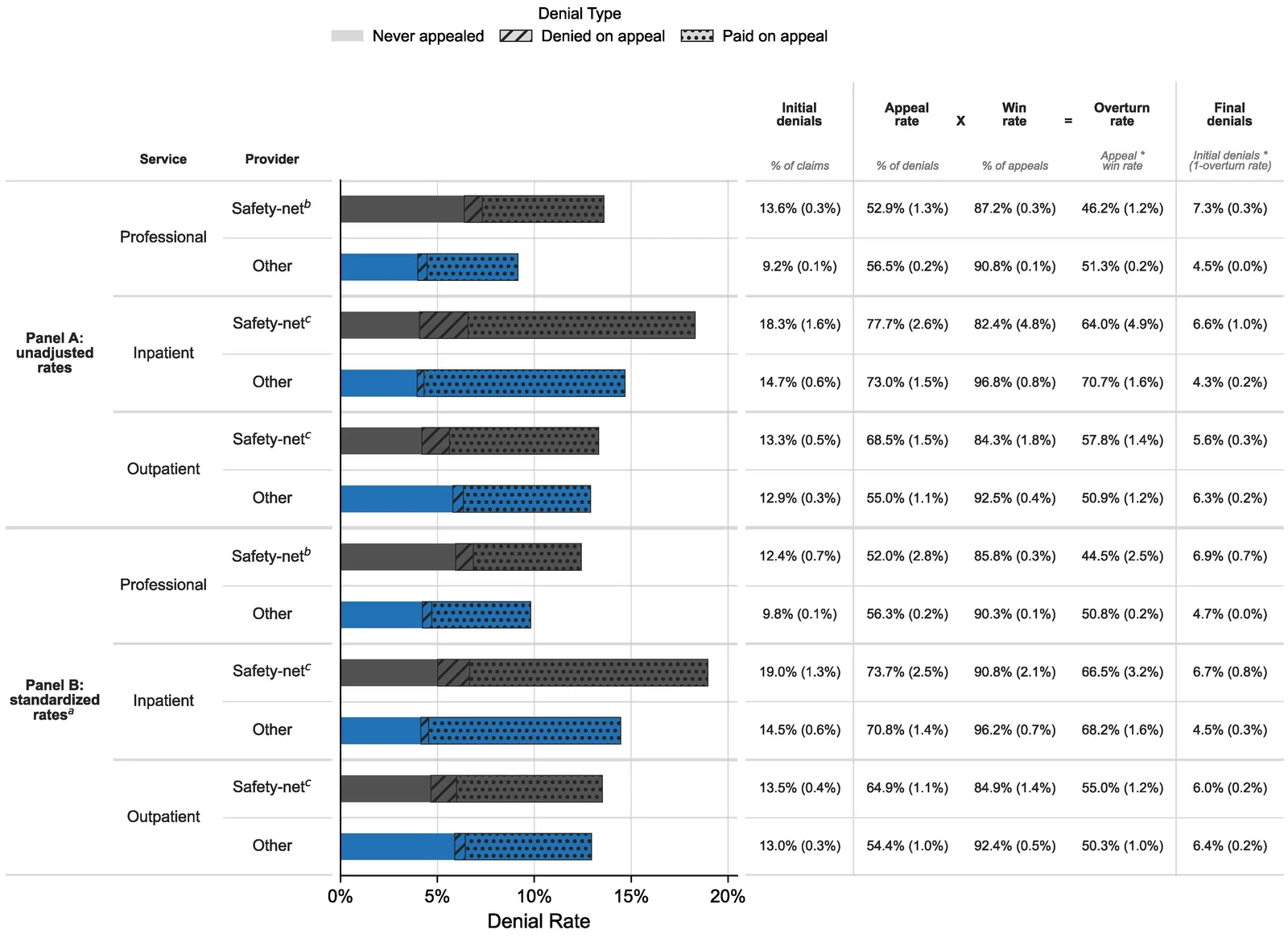
Denial rates by safety-net provider status. Source: Authors’ analysis of Inovalon claims dataset. Notes: Spending amounts calculated based on Traditional Medicare prices. ^a^Standardized rates were weighted to reflect the national payer mix for all providers. ^b^Safety-net professionals were those in the highest quintile for dual-eligibles as a share of Medicare patients. ^c^Safety-net hospitals were those in the highest quintile of Disproportionate Share Hospital Patient Percentage.

For professional claims, initial denials for safety-net providers were 13.6% vs 9.2% for nonsafety-net providers (Figure 2A). Safety-net providers also had a lower overturn rate (46.2% vs 51.3%), resulting in final denials of 7.3% vs 4.5%. Findings across the entire spectrum of providers’ dual-share were consistent and displayed a monotonic relationship. We present these data for all 5 quintiles of providers’ dual-share in Appendix Figure S1.

Inpatient claims followed a similar pattern. Initial denials for safety-net providers were 18.3% vs 14.7% for nonsafety-net providers, and safety-net providers also had a lower overturn rate (64.0% vs 70.7%), resulting in final denials of 6.6% vs 4.3%. For outpatient claims, initial denials for safety-net providers were again higher at 13.3% vs 12.9%. But due to a higher overturn rate (57.8% vs 50.9%), safety-net providers had a lower final denial rate (5.6 vs 6.3).

The findings were similar after standardizing for payer mix (Figure 2B). For professional claims, where Medicaid had the highest denial rate, reducing the weight of Medicaid through standardization brought safety-net providers somewhat closer in line with nonsafety-net providers, but changes were negligible for inpatient and outpatient services.

## Discussion

In this analysis of claim denials representing a large and diverse sample of insurers across the United States, we found that claim denials disproportionately impacted safety-net hospitals and physicians. They faced higher initial denial rates compared to other providers across all service types and had higher final denial rates for professional and inpatient services. The persistence of these differences after standardizing payer mix suggests that though some of these results reflect a higher proportion of Medicaid payers, safety-net providers also received more denials within some or all payer segments, independent of their payer mix.

Our study on the provider-level patterns of claim denials adds to a recent literature showing that denials are more common for socioeconomically disadvantaged individuals and the providers that serve them.^12,13^ The role of providers is important for research and policy discussions about claim denials because providers have a financial stake in claim decisions and can also influence the outcomes of those decisions.

Four hypotheses could explain the disparities. First, safety-net providers may have less capacity for revenue cycle management. Operating on thinner margins with fewer administrative resources, these providers may be more prone to documentation and coding errors that trigger denials and may be less able to invest in the appeals infrastructure needed to overturn them. This could lead to an adverse, reinforcing cycle where limited resources impact the ability to invest in revenue cycle and appeals processes, which in turn lead to higher denial rates and lower revenue, which in turn exacerbates the lack of funds for improving revenue collection.

Second, high denial rates could reflect safety-net providers’ poor bargaining leverage. Public statements from health systems reveal explicitly that denial rates and their drivers are indeed considerations in contract negotiations.^14^ Empirical work confirms that providers respond to denial rates as a contracting lever similar to payment rates.^11^ Provider–payer agreements vary widely on terms that can affect denial rates, such as timely appeal deadlines, site of care restrictions, medical necessity policies, and provider gold-carding (the practice of exempting some providers from prior authorization requirements).^21-23^ Just as safety-net providers have less leverage to negotiate favorable payment rates, they may also have less leverage to negotiate favorable terms and conditions.

Third, the nature of insurance coverage for safety-net providers’ patients may lead to more denials. For example, safety-net providers serve more patients that are dually eligible for Medicaid and Medicare and sometimes require complex coordination of benefits. Also, patients tend to churn in and out of Medicaid coverage more than other types of insurance, meaning coverage gaps and outdated coverage information are more common. These factors may result in more denials independently from billing practices.

Finally, higher denial rates could reflect differences in billing or clinical practices, including more aggressive coding, delivery of low-value or noncovered services, or, in some cases, noncompliant or fraudulent billing. Direct evidence comparing billing and clinical practices between safety-net and other providers is limited, and the available evidence does not support strong conclusions in either direction. Future research should explore the root causes of these disparities, ideally using data on the reasons for denials.

It is important for policymakers to consider these disparities as they debate the tradeoffs involved in claim denials. Although denials impose revenue loss and administrative costs on providers, they also may encourage better documentation and care processes and discourage wasteful care. If safety-net providers’ higher denial rates are mostly caused by less effective revenue cycle management or less leverage with health plans, then the incremental denials impose administrative costs without offsetting reductions in low-value care. Policy measures to reduce disparities in denials would be net positive, eliminating the administrative waste and resulting in a transfer from health plans to safety-net providers. In this case, policy should focus on helping safety-net providers improve revenue cycle operations so that more claims clear the “quality bar” for approval. Policymakers could also regulate the contractual rules around denials negotiated by providers and health plans to ensure the same “quality bar” is consistently applied to different providers.

## Conclusion

This study found meaningful variation in claim denial burdens across payers and provider types. Safety-net providers, for whom government payers comprise a much higher percent of revenue, faced higher initial denial rates for all service types compounded by lower rates of successful appeals for professional and inpatient claims. These findings suggest that the financial and administrative burdens of claim denials fall disproportionately on the providers least equipped to absorb them: providers serving low-income and elderly patients. As policymakers contemplate reforms to the denial and appeals process, they should consider how the burden of denials is distributed across the provider landscape.

## Supplementary Material

qxag206_Supplementary_Data
