# Supplementary material for: Variation in medical claim denials: safety-net providers are hardest hit
Source: Health Aff Sch. 2026 Aug 7;4(8):qxag206. doi: 10.1093/haschl/qxag206 (PMC13492083; doi:10.1093/haschl/qxag206)
Supplement: Supplementary file 1 [file qxag206_supplementary_data.zip › Denials_ms_appendix.docx]

**Appendix Table 1: Sample construction**

| *Number of claims (% of prior step) at each filtering stage.* | |  |  |  |
| --- | --- | --- | --- | --- |
|  |  |  |  |  |
| **Filtering step** | **Commercial** | **Medicaid** | **Medicare Advantage** | **Total** |
| ***Panel A. All claims*** |  |  |  |  |
| **Initial claims** | **285,973,558** | **177,143,914** | **66,320,378** | **529,437,850** |
| Missing claim status codes | 18,974,440 (6.6%) | 9,680,157 (5.5%) | 25,270,955 (38.1%) | 53,925,552 (10.2%) |
| Price < $1 / non-payment codes | 30,492,157 (11.4%) | 40,969,914 (24.5%) | 9,440,732 (23.0%) | 80,902,803 (17.0%) |
| **Complete cases** | **236,506,961** | **126,493,843** | **31,608,691** | **394,609,495** |
| Below provider volume threshold | 18,394,480 (7.8%) | 9,177,247 (7.3%) | 6,396,634 (20.2%) | 33,968,361 (8.6%) |
| No match with secondary data | 76,326,125 (35.0%) | 46,907,900 (40.0%) | 3,216,618 (12.8%) | 126,450,643 (35.1%) |
| **Final sample** | **141,786,356** | **70,408,696** | **21,995,439** | **234,190,491** |
| ***Panel B. Professional claims*** |  |  |  |  |
| **Initial claims** | **260,585,782** | **153,834,600** | **60,471,820** | **474,892,202** |
| Missing claim status codes | 16,958,654 (6.5%) | 9,233,751 (6.0%) | 23,410,979 (38.7%) | 49,603,384 (10.4%) |
| Price < $1 / non-payment codes | 30,491,227 (12.5%) | 40,969,641 (28.3%) | 9,438,395 (25.5%) | 80,899,263 (19.0%) |
| **Complete cases** | **213,135,901** | **103,631,208** | **27,622,446** | **344,389,555** |
| Below provider volume threshold | 17,615,847 (8.3%) | 8,261,461 (8.0%) | 5,978,676 (21.6%) | 31,855,984 (9.2%) |
| No match with Medicare NPI | 62,524,336 (32.0%) | 33,730,280 (35.4%) | 1,829,414 (8.5%) | 98,084,030 (31.4%) |
| Insufficient claims for NPI dual mix | 3,942,139 (3.0%) | 3,852,081 (6.2%) | 225,236 (1.1%) | 8,019,456 (3.7%) |
| **Final sample** | **129,053,579** | **57,787,386** | **19,589,120** | **206,430,085** |
| ***Panel C. Inpatient claims*** |  |  |  |  |
| **Initial claims** | **591,825** | **113,036** | **126,072** | **830,933** |
| Missing claim status codes | 53,596 (9.1%) | 2,866 (2.5%) | 41,400 (32.8%) | 97,862 (11.8%) |
| Price < $1 / non-payment codes | 930 (0.2%) | 271 (0.2%) | 2,337 (2.8%) | 3,538 (0.5%) |
| **Complete cases** | **537,299** | **109,899** | **82,335** | **729,533** |
| Below provider volume threshold | 22,945 (4.3%) | 6,459 (5.9%) | 5,414 (6.6%) | 34,818 (4.8%) |
| No match with AHRQ hospital | 154,024 (29.9%) | 8,236 (8.0%) | 13,310 (17.3%) | 175,570 (25.3%) |
| AHRQ hospital doesn't report DSH status | 15,286 (4.2%) | 1,895 (2.0%) | 2,691 (4.2%) | 19,872 (3.8%) |
| **Final sample** | **345,044** | **93,309** | **60,920** | **499,273** |
| ***Panel D. Outpatient claims*** |  |  |  |  |
| **Initial claims** | **24,795,951** | **23,196,278** | **5,722,486** | **53,714,715** |
| Missing claim status codes | 1,962,190 (7.9%) | 443,540 (1.9%) | 1,818,576 (31.8%) | 4,224,306 (7.9%) |
| Price < $1 / non-payment codes | 0 (0.0%) | 2 (0.0%) | 0 (0.0%) | 2 (0.0%) |
| **Complete cases** | **22,833,761** | **22,752,736** | **3,903,910** | **49,490,407** |
| Below provider volume threshold | 755,688 (3.3%) | 909,327 (4.0%) | 412,544 (10.6%) | 2,077,559 (4.2%) |
| No match with AHRQ hospital | 9,114,480 (41.3%) | 8,614,292 (39.4%) | 1,010,492 (28.9%) | 18,739,264 (39.5%) |
| AHRQ hospital doesn't report DSH status | 575,860 (4.4%) | 701,116 (5.3%) | 135,475 (5.5%) | 1,412,451 (4.9%) |
| **Final sample** | **12,387,733** | **12,528,001** | **2,345,399** | **27,261,133** |

**Appendix Figure 1: Professional Denial Rates by Provider Dual-Share Quintile**
